# Supplementary material for: Parental considerations about their childs’ mental health: Validating the German adaptation of the Parental Reflective Functioning Questionnaire
Source: PLoS One. 2024 Dec 4;19(12):e0314074. doi: 10.1371/journal.pone.0314074 (PMC11616854; doi:10.1371/journal.pone.0314074)
Supplement: S1 File — (DOCX) [file pone.0314074.s001.docx]

# SUPPLEMENTARY MATERIAL to “Parental Considerations About Their Childs’ Mental Health: Validating the German Adaptation of the Parental Reflective Functioning Questionnaire”

Andreas S. Wildner^1^, Su Mevsim Küçükakyüz^1^, Anton K. G. Marx^1^, Tobias Nolte^2^,

Corinna Reck^1^, Peter Fonagy^2^, Patrick Luyten^2^, Alexandra von Tettenborn^1^, Mitho

Müller^1^, Anna-Lena Zietlow^3^, and Christian F. J. Woll-Weber^1,4^

^1^Clinical Psychology of Childhood and Adolescence & Counseling Psychology

Ludwig-Maximilians-Universität, Munich, Germany

^2^Clinical, Education, & Health Psychology, Division of Psychology and Language Sciences,

Psychoanalysis Unit, University College London, UK

^3^Clinical Child and Adolescence Psychology, Institute of Clinical Psychology and

Psychotherapy, Technische Universität Dresden, Germany

^4^Clinical Child and Adolescence Psychology and Psychotherapy, Freie Universität Berlin, Germany

# Author Note

*Correspondence concerning this article should be addressed to Andreas S. Wildner, Department of Psychology, Clinical Psychology of Children and Adolescents Ludwig-Maximilians-Universität, Leopoldstr. 13, 80802 Munich, Germany. E-mail: andreas.wildner@psy.lmu.de

**SUPPLEMENTARY MATERIAL to “Parental Considerations About Their Childs’ Mental Health: Validating the German Adaptation of the Parental Reflective Functioning Questionnaire”**

# S1 Difference in Factor Structure between Different Adaptations of the PRFQ

In the validation for the Portuguese translation of the PRFQ, all subscales correlated significantly [1]. Another study from Canada also found all factors to correlate to a certain degree [2], same as a validation for the Italian translation [3], though it has to be mentioned that neither the Canadian nor the Italian study reported *p*-values for the factor correlations and the correlations overall ranged from small to large in effect size (*r* ranging from -.26 to .37 and -.51 to .10 respectively). A Korean validation study found that when using a three factor model, all factors were also correlated, but considerably stronger than in the Canadian or Portuguese studies (*r* ranging from -.83 to .60) [4]. However, the three factor model of the Korean version had a bad model fit overall, leading the authors to propose a five factor model using exploratory factor analysis, with mostly the items for PM migrated to the two new factors. A validation study of the Finish translation came up with a PRFQ version consisting of 14 items, loading onto four factors, with PM and IC, CMS and the fourth factor labeled "Uncertainty of child mental states", and PM and the fourth factor correlated [5]. A study from Hungary concluded a two factor model was the best fit [6], while a Chinese study proposed a 12-item, three factor model [7].

Literature Cited

1. Moreira H, Fonseca A. Measuring Parental Reflective Functioning: Further Validation of the Parental Reflective Functioning Questionnaire in Portuguese Mothers of Infants and Young Children. Child Psychiatry & Human Development 2022:1–13.

2. Roo M de, Wong G, Rempel GR, Fraser SN. Advancing Optimal Development in Children: Examining the Construct Validity of a Parent Reflective Functioning Questionnaire. JMIR Pediatrics and Parenting 2019; 2(1).

3. Pazzagli C, Delvecchio E, Raspa V, Mazzeschi C, Luyten P. The Parental Reflective Functioning Questionnaire in Mothers and Fathers of School-Aged Children. Journal of Child and Family Studies 2017; 27:80–90.

4. Lee Y, Meins E, Larkin F. Translation and preliminary validation of a Korean version of the parental reflective functioning questionnaire. Infant mental health journal 2021; 42:47–59.

5. Salo SJ, Pajulo M, Vinzce L, Raittila S, Sourander J, Kalland M. Parent Relationship Satisfaction and Reflective Functioning as Predictors of Emotional Availability and Infant Behavior. Journal of Child and Family Studies 2021; 30:1214–28.

6. Szabó B, Miklósi M, Boda M, Futó J. The adaptation of The parental reflective functioning questionnaire adolescent version to the Hungarian language and presentation of its psychometric characteristics. European Psychiatry 2022; 65(S1):207–8.

7. Ye P, Ju J, Zheng K, Dang J, Bian Y. Psychometric Evaluation of the Parental Reflective Functioning Questionnaire in Chinese Parents. Frontiers in Psychology 2022; 13.
