# Supplementary material for: Parental considerations about their childs’ mental health: Validating the German adaptation of the Parental Reflective Functioning Questionnaire
Source: PLoS One. 2024 Dec 4;19(12):e0314074. doi: 10.1371/journal.pone.0314074 (PMC11616854; doi:10.1371/journal.pone.0314074)
Supplement: S1 Fig — (DOCX) [file pone.0314074.s002.docx]

# SUPPLEMENTARY MATERIAL to “Parental Considerations About Their Childs’ Mental Health: Validating the German Adaptation of the Parental Reflective Functioning Questionnaire”

Andreas S. Wildner^1^, Su Mevsim Küçükakyüz^1^, Anton K. G. Marx^1^, Tobias Nolte^2^,

Corinna Reck^1^, Peter Fonagy^2^, Patrick Luyten^2^, Alexandra von Tettenborn^1^, Mitho

Müller^1^, Anna-Lena Zietlow^3^, and Christian F. J. Woll-Weber^1,4^

^1^Clinical Psychology of Childhood and Adolescence & Counseling Psychology

Ludwig-Maximilians-Universität, Munich, Germany

^2^Clinical, Education, & Health Psychology, Division of Psychology and Language Sciences,

Psychoanalysis Unit, University College London, UK

^3^Clinical Child and Adolescence Psychology, Institute of Clinical Psychology and

Psychotherapy, Technische Universität Dresden, Germany

^4^Clinical Child and Adolescence Psychology and Psychotherapy, Freie Universität Berlin, Germany

# Author Note

*Correspondence concerning this article should be addressed to Andreas S. Wildner, Department of Psychology, Clinical Psychology of Children and Adolescents Ludwig-Maximilians-Universität, Leopoldstr. 13, 80802 Munich, Germany. E-mail: andreas.wildner@psy.lmu.de

**SUPPLEMENTARY MATERIAL to “Parental Considerations About Their Childs’ Mental Health: Validating the German Adaptation of the Parental Reflective Functioning Questionnaire”**

# S2a Supplementary Fig 1. Histogram for the CMS subscale.


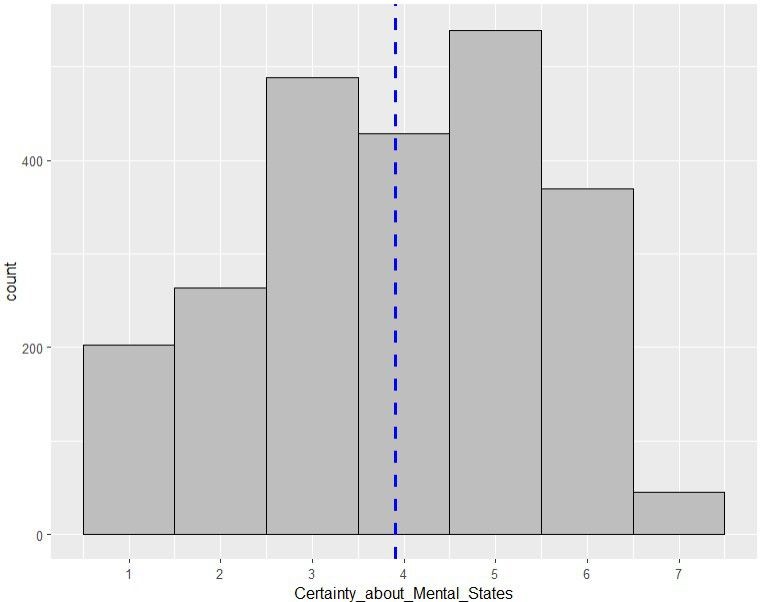


Figure available at [https://osf.io/j69wx/,](https://osf.io/j69wx/) under a CC-BY 4.0 license.

# S2b Supplementary Fig 2. Histogram for the IC subscale.


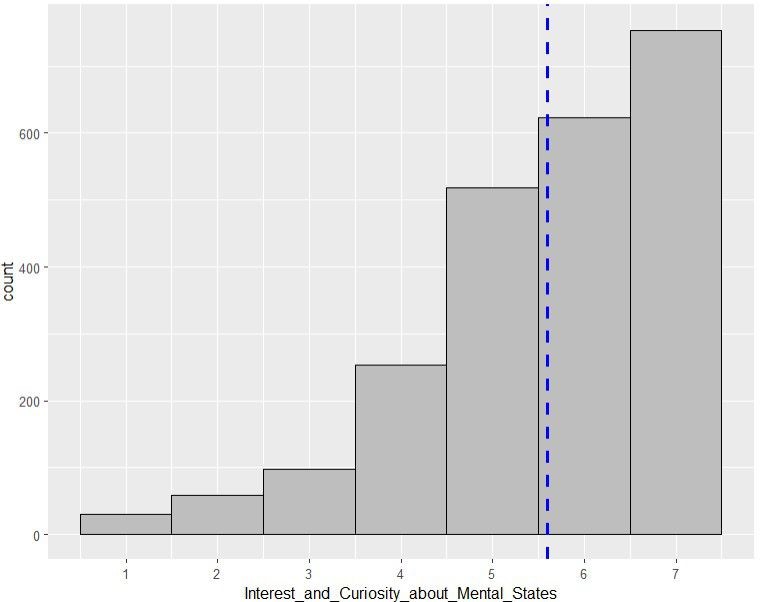


Figure available at [https://osf.io/j69wx/,](https://osf.io/j69wx/) under a CC-BY 4.0 license.

# S2c Supplementary Fig 3. Histogram for the PM subscale.


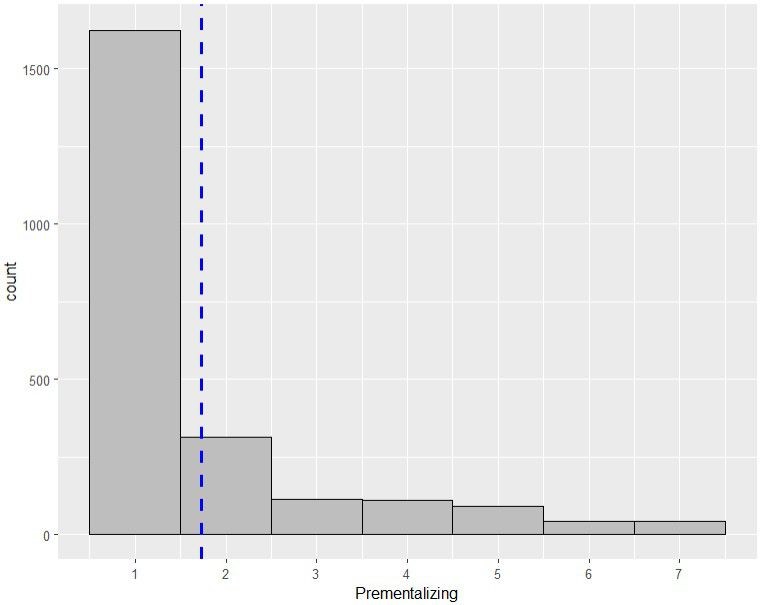


Figure available at [https://osf.io/j69wx/,](https://osf.io/j69wx/) under a CC-BY 4.0 license.
