# Supplementary material for: Parental considerations about their childs’ mental health: Validating the German adaptation of the Parental Reflective Functioning Questionnaire
Source: PLoS One. 2024 Dec 4;19(12):e0314074. doi: 10.1371/journal.pone.0314074 (PMC11616854; doi:10.1371/journal.pone.0314074)
Supplement: S1 Table — (DOCX) [file pone.0314074.s003.docx]

# SUPPLEMENTARY MATERIAL to “Parental Considerations About Their Childs’ Mental Health: Validating the German Adaptation of the Parental Reflective Functioning Questionnaire”

Andreas S. Wildner^1^, Su Mevsim Küçükakyüz^1^, Anton K. G. Marx^1^, Tobias Nolte^2^,

Corinna Reck^1^, Peter Fonagy^2^, Patrick Luyten^2^, Alexandra von Tettenborn^1^, Mitho

Müller^1^, Anna-Lena Zietlow^3^, and Christian F. J. Woll-Weber^1,4^

^1^Clinical Psychology of Childhood and Adolescence & Counseling Psychology

Ludwig-Maximilians-Universität, Munich, Germany

^2^Clinical, Education, & Health Psychology, Division of Psychology and Language Sciences,

Psychoanalysis Unit, University College London, UK

^3^Clinical Child and Adolescence Psychology, Institute of Clinical Psychology and

Psychotherapy, Technische Universität Dresden, Germany

^4^Clinical Child and Adolescence Psychology and Psychotherapy, Freie Universität Berlin, Germany

# Author Note

*Correspondence concerning this article should be addressed to Andreas S. Wildner, Department of Psychology, Clinical Psychology of Children and Adolescents Ludwig-Maximilians-Universität, Leopoldstr. 13, 80802 Munich, Germany. E-mail: andreas.wildner@psy.lmu.de

**SUPPLEMENTARY MATERIAL to “Parental Considerations About Their Childs’ Mental Health: Validating the German Adaptation of the Parental Reflective Functioning Questionnaire”**

# S3 Predicted Correlations and Hypothesis for Concurrent Validity

H3.1: The following significant (*p ≤* .05) correlations between the Epistemic Trust Mistrust and Credulity Questionnaire are expected: negative correlation between CMS and Mistrust and Credulity (H3.1.1), positive correlation between PM and Mistrust and Credulity (H3.1.3), negative correlation between PM and epistemic trust (H3.1.4). The following non-significant (n.s., *p* > .05) or negligible (*r ≤* .10) correlation are expected; n.s. correlation between CMS and epistemic trust (H3.1.2), negligible, neg correlation between IC and Mistrust and Credulity (H3.1.5).

H3.2: Correlations with the Perceived Stress Scale The following significant correlation is expected: positive correlation between PM and perceived stress (H3.2.1). The following n.s. correlation or negligible correlation are expected: n.s. correlation between IC and perceived stress (H3.2.2), and negligible correlation between CMS and perceived stress (H3.2.3).

H3.3: Correlations with the Edinburgh Postnatal Depression Scale The following significant correlation is expected: positive correlation between PM and postpartum depressiveness

(H3.3.1). The following n.s. correlation or negligible correlation are expected: n.s. correlation between IC and postpartum depressiveness (H3.3.2), positive, negligible correlation between CMS and postpartum depressiveness (H3.3.3).

H3.4: Correlations with demographic features The following significant correlations are expected: neg. correlation between level of education and PM (H3.4.3), positive correlation between relationship satisfaction and CMS and IC, negative cor between relationship satisfaction and PM (H3.4.4), positive correlation between changes in the working environment and PM (H3.4.5). The following n.s. correlations or negligible correlations are expected: n.s. correlation between parents’ age and PM (H3.4.1), n.s. cor between all three

PRFQ subscales and childs age (H3.4.2).

# S3 Supplementary Table 1. Assumed Correlations of Hypothesis Section 3

| Questionnaire | CMS | IC | PM |
| --- | --- | --- | --- |
| Trust (ETMCQ) | N.s. | N.s. | Neg. Cor. |
| Mistrust (ETMCQ) | Neg. Cor. | Negligible | Pos. Cor. |
| Credulity (ETMCQ) | Neg. Cor. | Negligible | Pos. Cor. |
| Perceived Stress (PSS) | Negligible | N.s. | Pos. Cor. |
| Postpartum Depression (EPDS) | Negligible | N.s. | Pos. Cor. |
| Age of Parents | N.s. | N.s. | Negligible |
| Age of Child | N.s. | N.s. | N.s. |
| Level of Education | N.s. | N.s. | Neg. Cor. |
| Relationship Satisfaction (PFB-K) | Pos. Cor. | Pos. Cor. | Neg. Cor. |
| Changes in the Working Environment | N.s. | N.s. | Pos. Cor. |

Assumed relationships were based on Luyten et al. [1] and Campbell et al. [2].

Abbreviations: Non-significant (N.s.), negative correlation (Neg. Cor.), positive correlation (Pos. Cor.).

Literature Cited

1. Luyten P, Mayes LC, Nijssens L, Fonagy P. The parental reflective functioning questionnaire: Development and preliminary validation. PLoS ONE 2017; 12(5).

2. Campbell C, Tanzer M, Saunders R, Booker T, Allison E, Li E et al. Development and validation of a self-report measure of epistemic trust. PLoS ONE 2021; 16(4).
