# Supplementary material for: Parental considerations about their childs’ mental health: Validating the German adaptation of the Parental Reflective Functioning Questionnaire
Source: PLoS One. 2024 Dec 4;19(12):e0314074. doi: 10.1371/journal.pone.0314074 (PMC11616854; doi:10.1371/journal.pone.0314074)
Supplement: S2 Table — (DOCX) [file pone.0314074.s004.docx]

# SUPPLEMENTARY MATERIAL to “Parental Considerations About Their Childs’ Mental Health: Validating the German Adaptation of the Parental Reflective Functioning Questionnaire”

Andreas S. Wildner^1^, Su Mevsim Küçükakyüz^1^, Anton K. G. Marx^1^, Tobias Nolte^2^,

Corinna Reck^1^, Peter Fonagy^2^, Patrick Luyten^2^, Alexandra von Tettenborn^1^, Mitho

Müller^1^, Anna-Lena Zietlow^3^, and Christian F. J. Woll-Weber^1,4^

^1^Clinical Psychology of Childhood and Adolescence & Counseling Psychology

Ludwig-Maximilians-Universität, Munich, Germany

^2^Clinical, Education, & Health Psychology, Division of Psychology and Language Sciences,

Psychoanalysis Unit, University College London, UK

^3^Clinical Child and Adolescence Psychology, Institute of Clinical Psychology and

Psychotherapy, Technische Universität Dresden, Germany

^4^Clinical Child and Adolescence Psychology and Psychotherapy, Freie Universität Berlin, Germany

# Author Note

*Correspondence concerning this article should be addressed to Andreas S. Wildner, Department of Psychology, Clinical Psychology of Children and Adolescents Ludwig-Maximilians-Universität, Leopoldstr. 13, 80802 Munich, Germany. E-mail: andreas.wildner@psy.lmu.de

**SUPPLEMENTARY MATERIAL to “Parental Considerations About Their Childs’ Mental Health: Validating the German Adaptation of the Parental Reflective Functioning Questionnaire”**

**English and German PRFQ items**

# S4a Supplementary Table 2. Items of the CMS subscale of the PRFQ and their translations

| Items Used in Luyten et al. [1] | German Translation by Krink et al. [3] |
| --- | --- |
| I always know what my child wants. | Ich weiß immer, was mein Kind will. |
| I can completely read my child’s mind. | Ich kann die Gedanken meines Kindes vollständig lesen. |
| I can always predict what my child will do. | Ich kann immer voraussagen, was mein Kind tun wird. |
| I can sometimes misunderstand the reactions of my child. | Manchmal kann ich die Reaktionen meines Kindes missver-  stehen. |
| **I always know why I do what I do to my child.** | **Ich weiß immer genau, warum ich das mit meinem Kind tue, was ich tue.** |
| I always know why my child acts the way he or she does. | Ich weiß immer, warum sich mein Kind so benimmt, wie es das tut. |

Item 5 (in bold) has been removed for the German adaptation. All items were measured on a 7-point Likert scale.

# S4b Supplementary Table 3. Items of the IC subscale of the PRFQ and their translations

| Items Used in Luyten et al. [1] | German Translation by Krink et al. [3] |
| --- | --- |
| I like to think about the reasons behind the way my child behaves and feels. | Ich mag es, über die Gründe des Verhaltens und der Gefühle meines Kindes nachzudenken. |
| **I wonder a lot about what my child is thinking and**  **feeling.** | **Ich frage mich häufig, was mein Kind denkt und**  **fühlt.** |
| I am often curious to find out how my child feels. | Ich bin oft neugierig darauf, wie sich mein Kind wohl fühlen mag. |
| I try to see situations through the eyes of my child. | Ich versuche, Situationen durch die Augen meines Kindes zu sehen. |
| I try to understand the reasons why my child misbehaves. | Ich versuche den Grund zu verstehen, warum sich mein Kind nicht benimmt. |
| I believe there is no point in trying to guess what my child  feels. | Ich glaube, es bringt nichts zu erraten, wie sich mein Kind wohl fühlen mag. |

Item 2 (in bold) has been removed for the German adaptation. All items were measured on a 7-point Likert scale.

# S4c Supplementary Table 4. Items of the PM subscale of the PRFQ and their translations

| Items Used in Luyten et al.[1] | German Translation by Krink et al. [3] |
| --- | --- |
| The only time I’m certain my child loves me is when he or she is smiling at me. | Nur wenn mein Kind mich anlächelt, bin ich mir sicher, dass es mich liebt. |
| My child cries around strangers to embarrass me. | Mein Kind weint vor Fremden, um mich in Verlegenheit zu  bringen. |
| I find it hard to actively participate in make believe play with my child. | Es fällt mir schwer, mich an Phantasie-Spielen mit meinem Kind aktiv zu beteiligen. |
| My child sometimes gets sick to keep me from doing what I want to do. | Manchmal wird mein Kind krank, um mich daran zu hindern das zu tun, was ich möchte. |
| When my child is fussy he or she does that just to annoy me. | Wenn mein Kind quengelig ist, tut es dies nur um mich zu  nerven. |
| Often, my child’s behavior is too confusing to bother figuring out. | Oft ist das Verhalten meines Kindes zu verwirrend, um sich die Mühe zu machen es zu verstehen. |

No items were removed in the German adaptation. All items were measured on a 7-point Likert scale.

# Questionnaire Items

The following section contains all used Questionnaires and their items except for the

PFB-K, which cannot be published due to copyright.

**ETMCQ**

# S4d Supplementary Table 5. Items of the German Translation of the Epistemic Trust, Mistrust, and Credulity Questionnaire [2]

| Item  Number | Items of the Epistemisches Vertrauen Fragebogen (EV-FB) |
| --- | --- |
| 1 | Ich frage üblicherweise andere um Rat, wenn ich persönliche Probleme habe. |
| 2 | Ich finde es leichter, Informationen zu vertrauen und aufzunehmen, wenn sie von jemandem stammen, der mich gut kennt. |
| 3 | Ich bevorzuge, Dinge im Internet selbst herauszufinden, statt andere um Informationen zu bitten. |
| 4 | Wenn mir jemand zeigen kann, dass etwas, was ich dachte, falsch ist, ändere ich normalerweise meine Meinung. |
| 5 | Es fällt mir schwer, Informationen von anderen zu vertrauen, mit denen ich nur wenig gemeinsam habe. |
| 6 | Ich befolge normalerweise keinen Ratschlag, den ich von anderen bekomme, selbst wenn ich denke, dass der Rat wahrscheinlich gut ist. |
| 7 | In der Vergangenheit habe ich falsch eingeschätzt, wem ich glauben kann, und bin deswegen ausgenutzt worden. |
| 8 | Wenn ich mich gut fühle, glaube ich Informationen, die mir jemand anderes gegeben hat, eher. |
| 9 | Ich habe oft das Gefühl, dass andere nicht verstehen, was ich will und brauche. |
| 10 | Ich werde oft für naiv gehalten, weil ich fast alles glaube, was andere mir erzählen. |
| 11 | Wenn ich mit verschiedenen Menschen spreche, kann ich mich leicht von dem überzeugen lassen, was sie sagen, auch wenn dies etwas anders ist, als das was ich vorher geglaubt habe. |
| 12 | Ein Gespräch mit Menschen, die mich schon lange kennen, kann mir helfen, neue Perspektiven über mich selbst zu entwickeln. |
| 13 | Ich finde es sehr nützlich, aus dem zu lernen, was andere mir über ihre Erfahrungen erzählen. |
| 14 | Wenn du dem, was andere dir erzählen, zu viel Glauben schenkst, bist du  leichter verletzbar. |
| 15 | Wenn mir jemand etwas erzählt, frage ich mich sofort, warum er mir das  jetzt erzählt. |
| 16 | Ich habe zu oft Ratschläge von den falschen Menschen angenommen. |
| 17 | Verschiedene Leute haben mir gesagt, dass ich zu leicht von anderen beeinflussbar bin. |
| 18 | Wenn ich nicht weiß, was ich tun soll, ist mein erster Impuls, jemanden zu fragen, dessen Meinung ich schätze. |

All items were measured on a 7-point Likert scale.

**Perceived Stress Scale**

# S4e Supplementary Table 6. Items of the German Translation of the Perceived Stress Scale by Reis et al. [4]

| Item  Number | Items of the Perceived Stress Scale |
| --- | --- |
| 1 | Wie oft waren Sie im letzten Monat aufgewühlt, weil etwas unerwartet  passiert ist? |
| 2 | Wie oft hatten Sie im letzten Monat das Gefühl, nicht in der Lage zu sein, die wichtigen Dinge in Ihrem Leben kontrollieren zu können? |
| 3 | Wie oft haben sie sich im letzten Monat nervös und gestresst gefühlt? |
| 4 | Wie oft waren Sie im letzten Monat zuversichtlich, dass Sie fähig sind, ihre persönlichen Probleme zu bewältigen? |
| 5 | Wie oft hatten Sie im letzten Monat das Gefühl, dass sich die Dinge zu Ihren Gunsten entwickeln? |
| 6 | Wie oft hatten Sie im letzten Monat den Eindruck, nicht all Ihren anstehenden Aufgaben gewachsen zu sein? |
| 7 | Wie oft waren Sie im letzten Monat in der Lage, ärgerliche Situationen in Ihrem Leben zu beeinflussen? |
| 8 | Wie oft hatten Sie im letzten Monat das Gefühl, alles im Griff zu haben? |
| 9 | Wie oft haben Sie sich im letzten Monat über Dinge geärgert, über die Sie keine Kontrolle hatten? |
| 10 | Wie oft hatten Sie im letzten Monat das Gefühl, dass sich so viele  Schwierigkeiten angehäuft haben, dass Sie diese nicht überwinden konnten? |

All items were measured on a 5-point Likert scale.

**Edinburgh Postnatal Depression Scale**

# S4f Supplementary Table 7. Items of the German Translation of the Edinburgh Postnatal Depression Scale by Bergant et al. [5]

| Item  Number | Items of the Edinburgh Postnatal Depression Scale |
| --- | --- |
| 1 | In den letzten sieben Tagen konnte ich lachen und das Leben von der sonnigen Seite sehen. |
| 2 | In den letzten sieben Tagen konnte ich mich so richtig auf etwas freuen |
| 3 | In den letzten sieben Tagen fühlte ich mich unnötigerweise schuldig, wenn etwas schief lief. |
| 4 | In den letzten sieben Tagen war ich ängstlich und besorgt aus nichtigen Gründen. |
| 5 | In den letzten sieben Tagen erschrak ich leicht, bzw. reagierte panisch aus unerfindlichen Gründen. |
| 6 | In den letzten sieben Tagen überforderten mich verschiedene Umstände. |
| 7 | In den letzten sieben Tagen war ich so unglücklich, dass ich nicht schlafen konnte. |
| 8 | In den letzten sieben Tagen habe ich mich traurig und schlecht gefühlt. |
| 9 | In den letzten sieben Tagen war ich so unglücklich, dass ich geweint habe. |
| 10 | In den letzten sieben Tagen überkam mich der Gedanke, mir selbst Schaden zuzufügen. |

All items were measured on a 4-point Likert scale.

Literature Cited

1. Luyten P, Mayes LC, Nijssens L, Fonagy P. The parental reflective functioning questionnaire: Development and preliminary validation. PLoS ONE 2017; 12(5).

2. Campbell C, Tanzer M, Saunders R, Booker T, Allison E, Li E et al. Development and validation of a self-report measure of epistemic trust. PLoS ONE 2021; 16(4).

3. Krink S, Muehlhan C, Luyten P, Romer G, Ramsauer B. Parental reflective functioning affects sensitivity to distress in mothers with postpartum depression. Journal of Child and Family Studies 2018; 27(5):1671–81.

4. Reis D, Lehr D, Heber E, Ebert DD. The German Version of the Perceived Stress Scale (PSS-10): Evaluation of Dimensionality, Validity, and Measurement Invariance With Exploratory and Confirmatory Bifactor Modeling. Assessment 2019; 26(7):1246–59.

5. Bergant A, Nguyen T, Heim K, Ulmer H, Dapunt O. Deutschsprachige Fassung und Validierung der »Edinburgh postnatal depression scale«. DMW-Deutsche Medizinische Wochenschrift 1998; 123(3):35–40.
