# Supplementary material for: Parental considerations about their childs’ mental health: Validating the German adaptation of the Parental Reflective Functioning Questionnaire
Source: PLoS One. 2024 Dec 4;19(12):e0314074. doi: 10.1371/journal.pone.0314074 (PMC11616854; doi:10.1371/journal.pone.0314074)
Supplement: S3 Table — (DOCX) [file pone.0314074.s005.docx]

# SUPPLEMENTARY MATERIAL to “Parental Considerations About Their Childs’ Mental Health: Validating the German Adaptation of the Parental Reflective Functioning Questionnaire”

Andreas S. Wildner^1^, Su Mevsim Küçükakyüz^1^, Anton K. G. Marx^1^, Tobias Nolte^2^,

Corinna Reck^1^, Peter Fonagy^2^, Patrick Luyten^2^, Alexandra von Tettenborn^1^, Mitho

Müller^1^, Anna-Lena Zietlow^3^, and Christian F. J. Woll-Weber^1,4^

^1^Clinical Psychology of Childhood and Adolescence & Counseling Psychology

Ludwig-Maximilians-Universität, Munich, Germany

^2^Clinical, Education, & Health Psychology, Division of Psychology and Language Sciences,

Psychoanalysis Unit, University College London, UK

^3^Clinical Child and Adolescence Psychology, Institute of Clinical Psychology and

Psychotherapy, Technische Universität Dresden, Germany

^4^Clinical Child and Adolescence Psychology and Psychotherapy, Freie Universität Berlin, Germany

# Author Note

*Correspondence concerning this article should be addressed to Andreas S. Wildner, Department of Psychology, Clinical Psychology of Children and Adolescents Ludwig-Maximilians-Universität, Leopoldstr. 13, 80802 Munich, Germany. E-mail: andreas.wildner@psy.lmu.de

**SUPPLEMENTARY MATERIAL to “Parental Considerations About Their Childs’ Mental Health: Validating the German Adaptation of the Parental Reflective Functioning Questionnaire”**

**Questionnaire Means**

# S5 Supplementary Table 8. Sample Means and Standard Deviations for all Questionnaires

| Questionnaire - Subscale | *M*(*SD*) |
| --- | --- |
| PRFQ - CMS | 3.91(1.07) |
| PRFQ - IC | 5.60(0.79) |
| PRFQ - PM | 1.72(0.58) |
| ETMCQ - Trust | 4.36(0.72) |
| ETMCQ - Mistrust | 3.76(0.77) |
| ETMCQ - Credulity | 3.86(0.77) |
| PSS | 19.37(7.02) |
| EPDS | 8.68(5.53) |
| PFB-K | 20.42(5.61) |
