# Supplementary material for: Parental considerations about their childs’ mental health: Validating the German adaptation of the Parental Reflective Functioning Questionnaire
Source: PLoS One. 2024 Dec 4;19(12):e0314074. doi: 10.1371/journal.pone.0314074 (PMC11616854; doi:10.1371/journal.pone.0314074)
Supplement: S4 Table — (DOCX) [file pone.0314074.s006.docx]

# SUPPLEMENTARY MATERIAL to “Parental Considerations About Their Childs’ Mental Health: Validating the German Adaptation of the Parental Reflective Functioning Questionnaire”

Andreas S. Wildner^1^, Su Mevsim Küçükakyüz^1^, Anton K. G. Marx^1^, Tobias Nolte^2^,

Corinna Reck^1^, Peter Fonagy^2^, Patrick Luyten^2^, Alexandra von Tettenborn^1^, Mitho

Müller^1^, Anna-Lena Zietlow^3^, and Christian F. J. Woll-Weber^1,4^

^1^Clinical Psychology of Childhood and Adolescence & Counseling Psychology

Ludwig-Maximilians-Universität, Munich, Germany

^2^Clinical, Education, & Health Psychology, Division of Psychology and Language Sciences,

Psychoanalysis Unit, University College London, UK

^3^Clinical Child and Adolescence Psychology, Institute of Clinical Psychology and

Psychotherapy, Technische Universität Dresden, Germany

^4^Clinical Child and Adolescence Psychology and Psychotherapy, Freie Universität Berlin, Germany

# Author Note

*Correspondence concerning this article should be addressed to Andreas S. Wildner, Department of Psychology, Clinical Psychology of Children and Adolescents Ludwig-Maximilians-Universität, Leopoldstr. 13, 80802 Munich, Germany. E-mail: andreas.wildner@psy.lmu.de

**SUPPLEMENTARY MATERIAL to “Parental Considerations About Their Childs’ Mental Health: Validating the German Adaptation of the Parental Reflective Functioning Questionnaire”**

**Overview of all Tested Models**

# S6 Supplementary Table 9. Overview of all tested models and their respective fit indices.

| # | Factors | Error Covar. | Items removed | *χ*2 | df | RMSEA | SRMR | TLI | CFI |
| --- | --- | --- | --- | --- | --- | --- | --- | --- | --- |
| 1 | 3 Factors, forced cor. | None | None | 307.75 | 132 | .082* | .090* | .712 | .751 |
| 2 | 3 Factors, forced cor. | IC2/IC3 | None | 272.63 | 131 | .073** | .084* | .767 | .800 |
| 3 | 3 Factors, forced cor. | IC2/IC3, PM2/PM4 | None | 232.48 | 153 | .064** | .077* | .821 | .848 |
| 4 | One global factor | None | None | 360.13 | 135 | .098 | .065 | .583 | .632 |
| 5 | 3 Factor, no forced cor. | None | IC2 | 240.49 | 116 | .073** | .082* | .774 | .807 |
| 6 | 3 Factor, no forced cor. | None | CMS5 | 232.15 | 116 | .070** | .081* | .774 | .808 |
| 7 | 3 Factor, no forced cor. | None, CMS5/6 | IC2, CMS5 | 170.02 | 101 | .057** | .074* | .850 | .839 |
| 8 | 3 Factor, no forced cor. | PM2/PM4, CMS5/6 | IC2, CMS5 | 127.65 | 100 | .038** | .065* | .933* | .944* |

*χ*^2^ = Chi-squared test statistic; *df* = Degrees of freedom; CFI = Comparative Fit Index; TLI = Tucker-Lewis Index; RMSEA = Root mean square error of approximation; SRMR = Standardized root mean square residual; *indicates good model fit; **indicates excellent model fit.
