# Supplementary material for: Parental considerations about their childs’ mental health: Validating the German adaptation of the Parental Reflective Functioning Questionnaire
Source: PLoS One. 2024 Dec 4;19(12):e0314074. doi: 10.1371/journal.pone.0314074 (PMC11616854; doi:10.1371/journal.pone.0314074)
Supplement: S5 Table — (DOCX) [file pone.0314074.s007.docx]

# SUPPLEMENTARY MATERIAL to “Parental Considerations About Their Childs’ Mental Health: Validating the German Adaptation of the Parental Reflective Functioning Questionnaire”

Andreas S. Wildner^1^, Su Mevsim Küçükakyüz^1^, Anton K. G. Marx^1^, Tobias Nolte^2^,

Corinna Reck^1^, Peter Fonagy^2^, Patrick Luyten^2^, Alexandra von Tettenborn^1^, Mitho

Müller^1^, Anna-Lena Zietlow^3^, and Christian F. J. Woll-Weber^1,4^

^1^Clinical Psychology of Childhood and Adolescence & Counseling Psychology

Ludwig-Maximilians-Universität, Munich, Germany

^2^Clinical, Education, & Health Psychology, Division of Psychology and Language Sciences,

Psychoanalysis Unit, University College London, UK

^3^Clinical Child and Adolescence Psychology, Institute of Clinical Psychology and

Psychotherapy, Technische Universität Dresden, Germany

^4^Clinical Child and Adolescence Psychology and Psychotherapy, Freie Universität Berlin, Germany

# Author Note

*Correspondence concerning this article should be addressed to Andreas S. Wildner, Department of Psychology, Clinical Psychology of Children and Adolescents Ludwig-Maximilians-Universität, Leopoldstr. 13, 80802 Munich, Germany. E-mail: andreas.wildner@psy.lmu.de

**SUPPLEMENTARY MATERIAL to “Parental Considerations About Their Childs’ Mental Health: Validating the German Adaptation of the Parental Reflective Functioning Questionnaire”**

**Factor Loadings**

# S7 Supplementary Table 10. Factor Loadings as obtained by EFA

| Item | CMS | IC | PM |
| --- | --- | --- | --- |
| CMS1 | .74 | .01 | .07 |
| CMS2 | .82 | .00 | .16 |
| CMS3 | .68 | .05 | .17 |
| CMS4 | .46 | -.25 | -.05 |
| **CMS5** | **.43** | **.01** | **-.30** |
| CMS6 | .74 | -.10 | -.11 |
| IC1 | .27 | .38 | -.25 |
| IC2 | -.23 | .69 | .09 |
| IC3 | -.02 | .71 | .06 |
| **IC4** | **.12** | **.35** | **-.35** |
| IC5 | .17 | .32 | -.05 |
| **IC6** | **.04** | **.13** | **-.23** |
| PM1 | .17 | .11 | .33 |
| PM2 | .10 | .10 | .43 |
| **PM3** | **-.15** | **-.05** | **.19** |
| PM4 | .17 | .02 | .46 |
| PM5 | .09 | .01 | .65 |
| PM6 | .00 | .04 | .53 |

Values obtained using EFA with a maximum likelihood estimator and promax rotation. Bold items were considered to cross-load or did not significantly load onto any factors.
