# Supplementary material for: Parental considerations about their childs’ mental health: Validating the German adaptation of the Parental Reflective Functioning Questionnaire
Source: PLoS One. 2024 Dec 4;19(12):e0314074. doi: 10.1371/journal.pone.0314074 (PMC11616854; doi:10.1371/journal.pone.0314074)
Supplement: Attachment — Submitted filename: PONE-D-24-14873.pdf [file pone.0314074.s008.pdf]

PLOS ONE

Parental Considerations About Their Childs' Mental Health: Validating the German Adaptation of the Parental Reflective Functioning Questionnaire

--Manuscript Draft--

**Manuscript Number:**	PONE-D-24-14873
**Article Type:**	Research Article
**Full Title:**	Parental Considerations About Their Childs' Mental Health: Validating the German Adaptation of the Parental Reflective Functioning Questionnaire
**Short Title:**	PRFQ: VALIDATION OF THE GERMAN ADAPTATION
**Corresponding Author:**	Andreas Wildner<br>Ludwig-Maximilians-Universität München: Ludwig-Maximilians-Universitat Munchen<br>München, GERMANY
**Keywords:**	Early parenthood, Mentalization, Reflective functioning, Self-report measure
**Abstract:**	Parental Reflective Functioning describes the parents' ability to view their child as motivated by mental states. The Parental Reflective Functioning Questionnaire (PRFQ) represents an 18-item and three-factor self-report measure. In the present study, we validate the German adaptation in a sample of n = 378 mothers. We used Confirmatory Factor Analysis with a cross-validation approach to assess model fit. Reliability was measured using Cronbach's α and McDonald's ω. Concurrent validity was assessed using correlations with relevant constructs. The three-factor structure of the original validation needed only minor modifications: two items had to be removed and one error covariance was added. The resulting 16-item questionnaire was successfully cross-validated. Correlations analyzed to assess concurrent validity were largely in accordance with the literature. Thus, while reliability could still be improved, the German 16-item version of the PRFQ represents a valid measure of parental reflective functioning as an important construct in developmental psychopathology.
**Order of Authors:**	Andreas Wildner
	Su Mevsim Küçükakyüz
	Anton K. G. Marx
	Tobias Nolte
	Corinna Reck
	Peter Fonagy
	Patrick Luyten
	Alexandra von Tettenborn
	Mitho Müller
	Anna-Lena Zietlow
	Christian F. J. Woll
**Additional Information:**	
**Question**	**Response**
**Financial Disclosure**<br><br>Enter a financial disclosure statement that describes the sources of funding for the work included in this submission. Review the submission guidelines for detailed requirements. View published research articles from *PLOS ONE* for specific	The author(s) received no specific funding for this work.

Powered by Editorial Manager® and ProduXion Manager® from Aries Systems Corporation

examples.<br><br>This statement is required for submission and **will appear in the published article** if the submission is accepted. Please make sure it is accurate.<br><br>**Funded studies**<br>Enter a statement with the following details:<br>• Initials of the authors who received each award<br>• Grant numbers awarded to each author<br>• The full name of each funder<br>• URL of each funder website<br>• Did the sponsors or funders play any role in the study design, data collection and analysis, decision to publish, or preparation of the manuscript?<br><br>Did you receive funding for this work?	
**Competing Interests**<br><br>Use the instructions below to enter a competing interest statement for this submission. On behalf of all authors, disclose any competing interests that could be perceived to bias this work—acknowledging all financial support and any other relevant financial or non-financial competing interests.<br><br>This statement is required for submission and **will appear in the published article** if the submission is accepted. Please make sure it is accurate and that any funding sources listed in your Funding Information later in the submission form are also declared in your Financial Disclosure statement.<br><br>View published research articles from *PLOS ONE* for specific examples.	The authors have declared that no competing interests exist.

**NO authors have competing interests**<br><br>Enter: *The authors have declared that no competing interests exist.*<br><br>**Authors with competing interests**<br><br>Enter competing interest details beginning with this statement:<br><br>*I have read the journal's policy and the authors of this manuscript have the following competing interests: [insert competing interests here]*<br><br>* typeset	
**Ethics Statement**<br><br>Enter an ethics statement for this submission. This statement is required if the study involved:<br><br>• Human participants<br>• Human specimens or tissue<br>• Vertebrate animals or cephalopods<br>• Vertebrate embryos or tissues<br>• Field research<br><br>Write "N/A" if the submission does not require an ethics statement.<br><br>General guidance is provided below. Consult the submission guidelines for detailed instructions. **Make sure that all information entered here is included in the Methods section of the manuscript.**	The study had been approved by the independent ethics committee of the medical faculty, Ruprecht-Karls-Universität, Heidelberg, in agreement with the ethics committee of<br>the Ludwig-Maximilians-Universität, Munich (vote: S-446/2017) and the declaration of Helsinki 2013, seventh revision.

**Format for specific study types**<br><br>**Human Subject Research (involving human participants and/or tissue)**<br>• Give the name of the institutional review board or ethics committee that approved the study<br>• Include the approval number and/or a statement indicating approval of this research<br>• Indicate the form of consent obtained (written/oral) or the reason that consent was not obtained (e.g. the data were analyzed anonymously)<br><br>**Animal Research (involving vertebrate animals, embryos or tissues)**<br>• Provide the name of the Institutional Animal Care and Use Committee (IACUC) or other relevant ethics board that reviewed the study protocol, and indicate whether they approved this research or granted a formal waiver of ethical approval<br>• Include an approval number if one was obtained<br>• If the study involved *non-human primates*, add *additional details* about animal welfare and steps taken to ameliorate suffering<br>• If anesthesia, euthanasia, or any kind of animal sacrifice is part of the study, include briefly which substances and/or methods were applied<br><br>**Field Research**<br><br>Include the following details if this study involves the collection of plant, animal, or other materials from a natural setting:<br>• Field permit number<br>• Name of the institution or relevant body that granted permission	
**Data Availability**<br><br>Authors are required to make all data underlying the findings described fully available, without restriction, and from the time of publication. PLOS allows rare exceptions to address legal and ethical concerns. See the PLOS Data Policy and FAQ for detailed information.	Yes - all data are fully available without restriction

A Data Availability Statement describing where the data can be found is required at submission. Your answers to this question constitute the Data Availability Statement and **will be published in the article**, if accepted.<br><br>**Important:** Stating 'data available on request from the author' is not sufficient. If your data are only available upon request, select 'No' for the first question and explain your exceptional situation in the text box.<br><br>Do the authors confirm that all data underlying the findings described in their manuscript are fully available without restriction?	
**Describe where the data may be found in full sentences. If you are copying our sample text, replace any instances of XXX with the appropriate details.**<br><br>• If the data are **held or will be held in a public repository**, include URLs, accession numbers or DOIs. If this information will only be available after acceptance, indicate this by ticking the box below. For example: *All XXX files are available from the XXX database (accession number(s) XXX, XXX.).*<br>• If the data are all contained **within the manuscript and/or Supporting Information files**, enter the following: *All relevant data are within the manuscript and its Supporting Information files.*<br>• If neither of these applies but you are able to provide **details of access elsewhere**, with or without limitations, please do so. For example:<br><br>*Data cannot be shared publicly because of [XXX]. Data are available from the XXX Institutional Data Access / Ethics Committee (contact via XXX) for researchers who meet the criteria for access to confidential data.*<br><br>*The data underlying the results presented in the study are available from (include the name of the third party*	The hypotheses, analysis plan, and cut-off criteria used in this study have been preregistered and are available along with the figures, analysis script, and all raw data necessary to reproduce the reported findings, under https://osf.io/j69wx/.

and contact information or URL).<br>• This text is appropriate if the data are owned by a third party and authors do not have permission to share the data.<br><br>* typeset	
Additional data availability information:	

Manuscript

Click here to access/download;Manuscript;PRFQ_German_Validation Plos

Parental Considerations About Their Childs' Mental Health: Validating the German Adaptation of the Parental Reflective Functioning Questionnaire

Andreas S. Wildner[1], Su Mevsim Küçükakyüz[1], Anton K. G. Marx[1], Tobias Nolte[2], Corinna Reck[1], Peter Fonagy[2], Patrick Luyten[2], Alexandra von Tettenborn[1], Mitho Müller[1], Anna-Lena Zietlow[3], and Christian F. J. Woll[1]

[1]Clinical Psychology of Childhood and Adolescence & Counseling Psychology

Ludwig-Maximilians-Universität, Munich, Germany

[2]Clinical, Education, & Health Psychology, Division of Psychology and Language Sciences, Psychoanalysis Unit, University College London, UK

[3]Clinical Child and Adolescence Psychology, Institute of Clinical Psychology and Psychotherapy, Technische Universität Dresden, Germany

Author Note

*Correspondence concerning this article should be addressed to Andreas S. Wildner, Department of Psychology, Clinical Psychology of Children and Adolescents Ludwig-Maximilians-Universität, Leopoldstr. 13, 80802 Munich, Germany. E-mail: andreas.wildner@psy.lmu.de

Abstract

Parental Reflective Functioning describes the parents' ability to view their child as motivated by mental states. The Parental Reflective Functioning Questionnaire (PRFQ) represents an 18-item and three-factor self-report measure. In the present study, we validate the German adaptation in a sample of $n = 378$ mothers. We used Confirmatory Factor Analysis with a cross-validation approach to assess model fit. Reliability was measured using Cronbach's α and McDonald's ω. Concurrent validity was assessed using correlations with relevant constructs. The three-factor structure of the original validation needed only minor modifications: two items had to be removed and one error covariance was added. The resulting 16-item questionnaire was successfully cross-validated. Correlations analyzed to assess concurrent validity were largely in accordance with the literature. Thus, while reliability could still be improved, the German 16-item version of the PRFQ represents a valid measure of parental reflective functioning as an important construct in developmental psychopathology.

Keywords: Early parenthood, Mentalization, Reflective functioning, Self-report measure

Parental Considerations About Their Childs' Mental Health: Validating the German Adaptation of the Parental Reflective Functioning Questionnaire

Introduction

PRF and its Relevance

Parental Reflective Functioning (PRF) relates to the parents' capacity to see their child as an intentional agent motivated by mental states, as well as the capacity to recognize how their own mental states affect their child (1). As such, PRF was described as an application of mentalizing, namely the capacity to consider both others' (other-mentalizing) and one's own intentional mental states (self-mentalizing, 2). PRF, as measured by the Parental Reflective Functioning Questionnaire (PRFQ, 3), is comprised of three dimensions. First, the Certainty about Mental States (CMS) captures the parents' awareness that mentalizing is inherently inferential and comes with some uncertainty. Second, the Interest and Curiosity about Mental States (IC) represents an attitude associated with more tolerance towards infants' distress (4, 5) and better regulation of the parents´ own mental states (3). Third, Pre-mentalizing (PM) aims to identify non-mentalizing modes, which would inhibit parents from entering the subjective world of their children (4) and can lead to misattributions of their children's actions (6).

PRF has been implicated in other relevant constructs such as children's development of Theory of Mind (7), epistemic trust (8), and attachment security (for an overview see 9). For instance, Meins et al. (10) found PRF to predict child attachment in the Strange Situation Test. Overall, high PRF seems to increase the likelihood of secure attachment in children (11) and can act as a protective factor for development in children with early adversities (12). On the

mother's side, low PRF increases occurrences of maladapted maternal behavior (13). This connection between PRF and attachment has been dubbed "loose coupling", describing the idea that secure attachment and emotional availability are not always, but often (14), associated with high PRF (15). In practice, indicators of functional PRF like IC and CMS are less often related to secure attachment than indicators of dysfunctional PRF, such as PM (3), which represents one of the main reasons to include the PM dimension in the PRFQ. One explanation for this well-documented connection between PRF and attachment security might be how PRF changes the way parents interact with their children. PRF leads to more parental sensitivity towards the child and also towards distress displayed by the child (16, 17). This, in turn, is an important predictor of the emergence of secure attachment (18).

The Measurement of PRF

Historically, the majority of available measures for PRF and Mentalizing were interviews (19), such as a specialized version of the Adult Attachment Interview (AAI) or the Parent Development Interview (PDI). While these measures allow for very detailed information and data analysis, their time-intensive nature makes them unsuitable for large-scale assessments (3). As such, the PRFQ is one of the first economic self-report scales of PRF (3). The PRFQ, as developed and validated by Luyten et al. (3), is an 18-item self-report scale. It consists of the three subscales CMS, IC, and PM each containing six items measured on a 7-point Likert scale.

While there has been some uncertainty regarding the factor structure of the PRFQ, Luyten et al. (3) proposed that their 18-item questionnaire comprises three factors, of which only CMS and IC are correlated. In their second study (3), this correlation was no longer significant. The model also contains a number of unspecified error covariances. To date, the

PRFQ has been validated in various countries, including Portugal, Canada, South Korea, China, Italy, Hungary, Finland, and Iran (20–28). Many of these studies found deviating factor structures, with factor counts ranging from two to five, and varying factor correlations, and as many as six items being removed. A more detailed discussion of these differences can be found in the online supplementary material.

The Current Study

The present study has two goals: First, we aim to replicate the factor model of the original PRFQ. Second, we aim to validate the German version of the PRFQ. This German version has been used first by Krink et al. (17), after translating the PRFQ from English to German and back by an English native speaker. However, this study only assessed the reliability as measured by Cronbach's α and, until today, this German adaptation has not been validated further (29). While some publications have used the validation of the original PRFQ to demonstrate sufficient construct validity for the German translation (e.g., 30), this is inadvisable since it needs to be assured that a translation is not only verbally correct but also transmits the intended meaning, tone, and cultural differences (31). To close this research gap, we aim to provide a full validation of the German translation of the PRFQ.

Hypotheses

The following hypotheses have been formulated based on previous research by Campbell et al. (32) and Luyten et al. (3).

Factor Structure

H1: The study assumes a replication of the model of Luyten et al. (3)– Study 1. The original model provides a good model fit (H1.1) and has the same significant factors and loadings as Luyten et al. (3, H1.2).

Reliability

H2: All scales and subscales prove a reliable measure as indicated by both a Cronbach's α and a McDonald's ω value of > .70.

Concurrent Validity

To test the concurrent validity, a total of 30 individual hypotheses were preregistered, which can be found in the online supplement (for an overview see Supplementary Table 1).

H3: We expect CMS, IC, and PM to be significantly correlated to relationship satisfaction. We expect CMS to be correlated with the Epistemic Trust Mistrust and Credulity Questionnaire (ETMCQ). We expect PM to be significantly correlated to the ETMCQ, perceived stress, postpartum depressiveness, education, and changes in the working environment due to COVID-19.

Method

Participants & Procedure

The sample for this study is part of a longitudinal project called CoviFam, as part of a larger longitudinal, observational project called COMPARE (33). In the CoviFam project, we investigate the well-being in a community sample of young families in the context of the COVID-19 pandemic. The initial recruiting for the CoviFam project took place from 06/05/2020 to

20/11/2020. All data used in this study (34) stems from the third measuring time point, which took place from 23/02/2022 to 06/05/2022. Participants were informed about the content of the study as well as their right to withdraw at any time. Afterwards, they gave their written, informed consent prior to filling out an online questionnaire at a time and place of their choosing. The target population were mothers of children aged 0 to 3 years, which were recruited for the study by various social media channels, medical institutions and professionals, and professional support organizations. The final sample size was N = 378, with 19 cases being removed due to missing data or IDs entered erroneously by the mothers, and 40 participants for indicating a gender other than female, to stick with the parameters used by Luyten et al. (3). The mothers' mean age was M = 35.79 (SD = 4.37, range = 19 - 50 yrs). The children's mean age was M = 43.44 months (SD = 13.32, range = 10.2 - 78.6). Further demographics can be found in Table 1.

Materials

The study consisted of an extensive online-based self-report questionnaire. Parental Reflective Functioning was measured via the PRFQ by Luyten et al. (3). The original PRFQ is an 18-item 3-factor self-report questionnaire using a 7-point Likert scale. The items of each subscale are averaged, yielding three mean values for each participant. The items used in the validation by Luyten et al. (3) as well as their translations for the present study can be found in Supplementary Tables 2-4.

Next to the PRFQ, the following variables and corresponding instruments were chosen to assess concurrent validity (see Supplementary Tables 5-7 for all instruments): The German translation of the (ETMCQ, 32), the German translation of the Perceived Stress Scale (PSS-10,

35), the German translation (36) of the Edinburgh Postnatal Depression Scale (EPDS-10, 37), and the shortened version of the Relationship Questionnaire ("Partnerschaftsfragebogen", PFB-K, 38). Means and standard deviations for all measures can be found in Supplementary Table 8. Additionally, the following additional demographic variables were obtained: Children’s and parents’ age, the level of secondary education achieved, and if or not the parents’ working environment had changed due to the COVID-19 pandemic.

Table 1. Participants’ Sociodemographic Characteristics

	n	**%**
Child’s gender	378	
Female	188	49.74
Number of children in the family	378	
1 child	138	37.51
2 children	182	48.15
3 children	47	12.43
4 children	9	2.38
5 children	2	0.53
Relationship status	378	
Married (living together)	319	84.39
Relationship (living together)	55	14.55
Relationship (not living together)	4	1.06
School degree	378	
German middle school diploma*	5	1.32
German Realschule diploma	38	10.05
German Fachabitur	49	12.96
German Abitur	286	75.66
Monthly net income	378	
0 - 1000 €	3	0.79
1000 - 2000 €	9	2.38
2000 - 3000 €	55	14.55
3000 - 5000 €	183	48.41
> 5000 €	128	33.86

Change in job situation due to the pandemic	378	
Working environment changed	146	38.62

n = sample size. *: German middle school = "Mittelschule" (Grade 5-9).

Analysis

The goal of this study was to confirm the factor structure of the original PRFQ via confirmatory factor analysis (CFA) or, alternatively, to propose a new factor structure. Models were assessed in reliability, measurement fit, and concurrent validity.

All data analyses were carried out in R Studio (39), utilizing the following packages: lavaan (40), tidyverse (41), eeptools (42), lubridate (43), psych (44), naniar (45), semPlot (46), coefficientalpha (47), Hmisc (48), moments (49), and e1071 (50). The study followed the recommendations of Jackson et al. (51) for the application of CFA. Little's MCAR test was used to inspect whether data was missing completely at random (MCAR).

Reliability was assessed using McDonald's Omega (ω, 52) and Cronbach's Alpha (α, 53). Values of > .70 were seen as indicative of a reliable scale (54). Next, CFA was run with the original factor model of the PRFQ (Study 1, 3). To assess model fit, the recommendations of Hu and Bentler (55) and Jackson et al. (51) were followed. We reported the χ^2 value despite its tendency to be overly sensitive to sample size (56) but relied on the Comparative Fit Index (CFI), Standardized Root Mean Square Residual (SRMR), Root Mean Square Error of Approximation (RMSEA), and Tucker-Lewis Index (TLI) to determine model fit. The cut-off values for good model fit were CFI > .90, TLI > .90, SRMR < .09, and RMSEA < .08. Cut-offs for excellent model fit were CFI > .95, TLI > .95, SRMR < .08, and RMSEA < .06 (55). A covariance

matrix was chosen as input matrix and a robust maximum likelihood (MLR) estimator was used.

The skewness and distribution were assessed. A parallel analysis using a 95th percentile criterion was run to inspect the suggested number of factors (57, 58). An exploratory factor analysis (EFA) with a maximum likelihood estimator, promax rotation, and the number of factors as suggested by parallel analysis was used to inspect item loadings. Next, the sample was randomly split into two equal groups for training and testing a new model via CFA. Using the training data set, a total of 8 models were tested using lavaan's cfa() function (40). The models incorporated varying factor correlations, residual item correlations, and deleted items (see Supplementary Table 9 for all tested models). We aimed to identify a model with at least an acceptable fit and having the least amount of modifications. After having discovered a working model, it was cross-validated using the testing data set. Finally, the reliability was once again calculated using Cronbach's α and McDonald's ω.

The concurrent validity was analyzed via correlation analyses with the ETMCQ, PSS, EPDS, age of child and parents, relationship satisfaction, education, and changes in the working environment. Effect sizes were presumed as small for $r > .10$, medium for $r > .30$, and $r > .50$ (59). An overview of the expected correlations can be seen in the online supplements. Additionally, we explored a bifactorial model. We employed a Schmid-Leiman transformation and an oblimin factor rotation, using the approach outlined in (60). This model added a general factor to the original model. To assess model fit, the Root Mean Squared Residuals (RMSR) was employed, with the same cut-off as the standardized RMSR, < .08 for good model fit and < .09 for excellent fit (55).

The standard $p < .05$ criterion was used to determine if factor loadings and correlations were significant. No further corrections were applied as all analyses contain different combinations of variables and a robust estimator was used. No transformations were applied.

Results

Analysis of Missing Data

The only missing data were the answers of 11 participants in the PFB-K, measuring relationship satisfaction. Little's MCAR test was non-significant ($\chi^2 = 66.0$, $df = 62$, $p = .340$), indicating that missing data was completely at random and list-wise deletion was applied.

Confirmatory Factor Analysis of the Original PRFQ Model

Using the original model for a CFA, all factors loaded significantly onto their respective items, and the IC and CMS subscale correlated significantly ($r = .48$, $p < .001$). However, the model showed poor fit (55): CFI = .80, TLI = .78, SRMR = .09, RMSEA = .07 (CI [.06, .08]), $\chi^2 = 374.62$ ($p < .001$). As such, the original model could not be replicated. The reliability assessment with Cronbach's α showed (1) $\alpha = .80$ for CMS, (2) $\alpha = .66$ for IC, and (3) $\alpha = .54$ for PM. The reliability as analyzed by McDonald's ω returned (1) $\omega = .88$ for CMS, (2) $\omega = .73$ for IC, and (3) $\omega = .61$ for PM. None of these values could be significantly improved by removing items. Further information on skew and distribution is available in the online supplement (for graphical representations see Supplementary Figures 1-3). Given that the original model did not hold, all results beyond this point are directly based on the new model we propose for the German PRFQ.

Model Adaptation for the German Translation

First, a parallel analysis was run to check the number of suggested factors. Both the parallel analysis and the scree plot suggested three factors, with Eigenvalues of 3.3 for factor 1, 1.3 for factor 2, and 0.8 for factor 3. Next, an EFA with the suggested three factors was used to inspect factor loadings of individual items (see Supplementary Table 10). The CMS factor explained 16% of variance with loadings ranging from .43 to .74, the IC factor 8% with loadings between .13 and .71, and the PM factor 9% with loadings ranging from .19 to .65.

The sample was randomly split in two groups. The first group was used to find a new model by testing a total of 8 CFA models (see the online supplement for an overview of the models). Ultimately, a model that provided good fit was found (see Figure 1). After removing the second item of the IC subscale, the fifth item of the CMS subscale, adding an error covariance between the second and fourth item of the PM scale, and allowing the factors to freely correlate, all fit indices indicated a good to excellent fitting model: CFI = .94, TLI = .93, SRMR = .07, RMSEA = .04 (CI [.01, .06]). The χ^2 value was 127.65 (p = .032). The IC factor correlated significantly with the CMS factor (r = .49, p < .001), as well as with the PM factor (r = -.59, p = .001). The correlation between CMS and PM was non-significant (r = -.23, p = .094). To cross validate the new 16-item model, the second half of the sample was then used to run another CFA. The fit indices showed good model fit again: CFI = .92, TLI = .90, SRMR = .07, RMSEA = .05 (CI [.03, .06]). The χ^2 value was 145.05 (p = .002). The reliability values of the subscales, as measured by Cronbach's α, pertaining to the new model, are as follows: 1. CMS: α = .79, 2. IC: α = .64, and 3. PM: α = .54. These values indicate a reliable CMS subscale. The reliability of the IC and PM subscale did not meet our reliability cutoff of α = .70. The reliability

values of the subscales, as measured by McDonald's ω, pertaining to the new model, are as follows: (1) CMS: ω = .84, (2) IC: ω = .69, and (3) PM: ω = .61. These values indicate a reliable CMS subscale. The reliability of both IC and PM fell short of our cut-off value of ω = .70.

Table 2. Correlational Analyses Assessing Concurrent Validity

Questionnaire	CMS	IC	PM
Trust (ETMCQ)	*N.s.*	*N.s.*	Neg. Cor.
	.01	*.03*	.12
	(.861)	*(.555)*	(.020)
Mistrust (ETMCQ)	Neg. Cor.	*Negligible*	*Pos. Cor.*
	-.01	*.10*	*.18*
	(.851)	*(.058)*	*(<.001)*
Credulity (ETMCQ)	Neg. Cor.	*Negligible*	*Pos. Cor.*
	.07	*.07*	*.11*
	(.163)	*(.188)*	*(.028)*
Perceived Stress (PSS)	Negligible	N.s.	*Pos. Cor.*
	-.23	-.12	*.27*
	(<.001)	(.022)	*(<.001)*
Postpartum Depression (EPDS)	*Negligible*	*N.s.*	*Pos. Cor.*
	-.07	*-.04*	*.26*
	(.161)	*(.454*	*(<.001)*
Age of Parents	*N.s.*	N.s.	*Negligible*
	-.06	-.14	*.05*
	(.285)	(.006)	*(.362)*
Age of Child	*N.s.*	N.s.	N.s.
	.00	-.10	.15
	(.924)	(.047)	(.003)
Level of Education	N.s.	*N.s.*	Neg. Cor.
	-.12	*.07*	-.10
	(.024)	*(.195)*	(.042)
Relationship Satisfaction (PFB-K)	*Pos. Cor.*	*Pos. Cor.*	Neg. Cor.
	.10	*.13*	-.09
	(.045)	*(.009)*	(.083)
Changes in the Working Environment	*N.s.*	*N.s.*	Pos. Cor.
	-.02	*.01*	-.01
	(.736)	*(.901)*	(.886)

The first line of each cell contains outcomes as predicted by the pre-registration (https://osf.io/j69wx/), with options being a predicted positive correlation (Pos. Cor.), negative correlation (Neg. Cor.), negligible meaning significant but $r \leq .10$, and finally not significant (N.s.). The second line contains the correlation coefficient, and lastly the third line contains the *p*-value. Italic correlations were in line with the predictions. Correlations which were assumed to be negligible but ended up being non-significant were still deemed correctly predicted.

Figure 1. Proposed Factor Model of the German PRFQ.

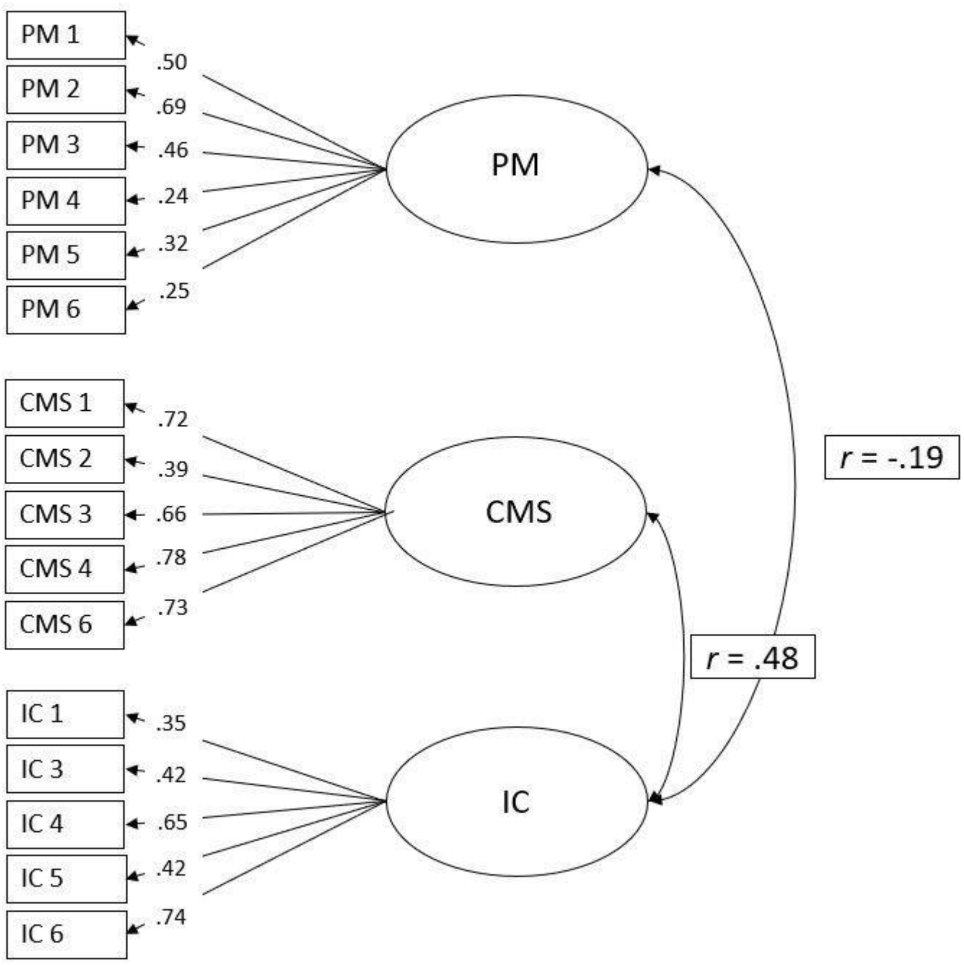

All estimates were statistically significant. Figure available at https://osf.io/j69wx/, under a CC-BY 4.0 license.

Bifactorial Model

The bifactorial model using the same 16-item architecture was run with the addition of a general factor. The model yielded an RMSR of .25, indicating a misspecified model. Hence, this approach was not further pursued.

Concurrent Validity

Summarizing the assessment of the concurrent validity, 18 out of 30 preregistered correlations were correctly anticipated. All incorrectly anticipated correlations were in the small effect range

(r = -.12 to .15). Only the correlation between CMS and perceived stress stood out, with an effect size of small to medium (r = -.23), indicating that individuals reporting higher levels of stress tended to struggle more with PRF. All correlations can be seen in Table 2.

Discussion

The overall goals of the present study were (1) to replicate the original PRFQ factor structure or (2) to propose a new model for the German PRFQ via exploratory means, as well as (3) to assess the reliability of the German PRFQ and (4) to analyze its concurrent validity.

Interpretation and Comparisons of the Results

Model Replication and Alternative Proposition. The original factor model of Luyten et al. (3) could not be replicated and the CFI, TLI, and SRMR indicated that the model was misspecified (55). Notably, given that Luyten et al. (3) had to add a number of not specified error covariances to achieve model fit, we were not able to test the exact same model. The remaining discussion will only address our newly proposed model for the German PRFQ.

Alternative Proposition of a new Model. Regarding a new model for the German PRFQ, parallel analyses suggested a three-factor model. A subsequent EFA with three factors was run, following the recommendations by Hair et al. (61) and considering factor loadings of .30 to be significant. For CMS, all items loaded significantly onto the subscale, along with item 5 cross-loading onto the PM subscale. For IC, items 1-5 loaded onto IC, item 4 additionally cross-loaded onto PM and item 6 did not significantly load onto any of the subscales. A number of other translations also had issues with factor loadings with item 6 (20, 27, 28). Lastly, for PM, all items but number 3 loaded on the subscale. The EFA showed all item loadings as intended, with cross-loadings being rare and if present only low in effect size. It was therefore concluded that

the EFA did not warrant modifications to the factor structure.

The new model demonstrated excellent fit as judged using RMSEA and SRMR and good fit using TLI and CFI (see 1). This model contained the same three factors, but allowed free correlation. The second item of the IC scale ("I wonder a lot about what my child is thinking and feeling.") and the fifth item of the CMS scale ("I always know why I do what I do to my child.") were removed. Lastly, we had to add one error covariance between the second and fourth PM items. Ideally, the latter measure would have been avoided, but since the items came from the same subscale and correlated, this was deemed acceptable. The χ^2 value was significant, indicating misspecification. However, given this metric's tendencies to dismiss adequate models in larger sample sizes (56), coupled with the results from the other four fit indices, we assumed our model to show good to excellent fit. In our cross-validation, RMSEA and SRMR again indicated excellent fit and TLI and CFI indicated good fit, which suggests no overfitting. This cross-validation together with our conservative approach with cut-off values further strengthens our proposed model and supports the original factor model (3).

Interestingly, the Finnish validation (23) also excluded item 2 from the IC subscale. A possible explanation might be its double-barreled wording ("I wonder a lot about what my child is thinking and feeling."), asking about both the cognitive component (thinking) and the affective component (feeling). Additionally, the German translation "Ich frage mich häufig..." might come across as more serious and obsessive as compared to the original English "I wonder a lot...". The fifth CMS item is the only item concerned with the mental state of the parent, not the child ("I always know why I do what I do to my child."). While theoretically still part of PRF, it is a clear difference from the other items. The Certainty About Mental States Questionnaire

(CAMSQ), a self-report measure designed to only assess CMS, also differentiates between self- and other-certainty, and achieves good reliability (62). The CAMSQ therefore gives empirical evidence that the two abilities of mentalizing about oneself and others are related, but not the same, and subsequently why the removal of this item improves fit. However, while the CAMSQ (62) only captures the dimension of certainty about mental states, which is also captured by the CMS subscale in the PRFQ, the PRFQ provides the possibility to capture two additional dimensions of PRF. Another issue could be the wording of the item "I always know why I do what I do to my child." in the German translation ("Ich weiß immer genau, warum ich das mit meinem Kind tue, was ich tue"), which changes the item from "i always know why..." to "i always know *exactly* why...". Thus, we advise that researchers should reassess this item after having removed the "genau" (= exactly) and then rerun the model to check if this change makes the item viable again. Looking back to the EFA results, this item was also the only one cross-loading in that particular subscale.

While the prospect of having a bifactorial model would have opened some exciting avenues for future research, the RMSR value that we obtained from our analysis could not support this modeling approach.

Reliability Analysis. Judging by Cronbach's α and McDonald's ω, the CMS subscale showed good reliability, while the reliabilities of the IC and PM subscale needs further assessment and improvement. These findings are still mostly on par with other validations of the PRFQ. While PM only barely made the cut-off of .70 in the original validation (α = .70), it was below the cut-off in the Italian, Korean, and Finnish validations (21, 23, 24), with the Italian version also struggling with the reliability of IC. In its first application (26), the 18-item version of the

German PRFQ struggled with reliability of the IC (α = .47) and PM (α = .52) subscale. On the other hand, the Portuguese (22) and Canadian validations (20) showed reliable measures of all three subscales.

Considering these results as well as those of prior studies, the internal consistencies of the IC and PM subscales appear to be an issue in most languages (63). A possible explanation for this might be found in the complexity of the construct, reflected in its measurement using complex coding of interviews and multi-factored self-report questionnaires. PRF and Mentalizing require both affective and cognitive processes (64), meaning that parts of these particular mental abilities rely on unconscious processes (16) which might not be easily captured by self-report, especially PM. Another consideration is social desirability, particularly with some very strongly worded PM items. This might partly explain the extreme skew of the PM subscale. However, this subscale has in part been developed for clinical risk samples. Given that we investigated a general, non-clinical sample, low average scores may be expected. Overall, the German PRFQ still shows acceptable performance in terms of reliability.

Concurrent Validity Analysis. The majority of our predictions about concurrent validity were in line with our results (18 out of 30 hypotheses tested). The negative correlation between CMS and perceived stress stood out. However, both the Canadian validation (20) and Luyten et al. (3) also showed that individuals reporting higher levels of stress also scored lower on PRF, however their effects remained insignificant. One possible reason for this might be the increase in stress during the COVID-19 pandemic, since high stress might impact mentalizing capacity (65, 66). Perceived stress increased by more than a standard deviation in our sample (67) compared to German mothers pre-pandemic (M = 22.29, SD = 3.43 vs. M = 13.07, SD = 6.08, 68). Therefore,

contextual effects on PRF cannot be ruled out. Another source for the deviation could be that, following the theory behind the CMS scale, it should be mapped non-linearly, since both exceedingly high and low values should be considered unfavorable (hyper- and hypomentalizing respectively). This mismatch between theory and practice might also cause artifacts in our correlation, though it would not explain why this has not been an issue for other validation studies of the PRFQ. Finally, taking all analyses into consideration, it can be assumed that the German PRFQ demonstrated adequate concurrent validity.

Limitations

The main limitations of the present study stem from its sample, mainly its high SES background. The majority of parents were in the two highest income brackets and also had the highest achievable school degree. For further validation, it would be desirable to also include lower SES backgrounds. Additionally, the data we used did not contain any information on ethnic identity. Second, the sample was highly motivated, since this was the third time they participated in the longitudinal study, meaning some self-selection has to be assumed. As a third limitation, the reliability of the presented questionnaire still poses an issue and requires further clarification.

Recommendations for Further Research

To increase reproducibility (69), we encourage the use of open science methods. The majority of other validations provided no preregistration or open data, which made replication significantly harder. By providing these additional materials, subsequent studies have a stronger foundation to build upon.

Another issue is the scaling of the CMS subscale. The IC and PM subscales are mostly modeled in a linear fashion. However, as already discussed, CMS might follow an inverted U-shape function because of hyper- and hypomentalizing being situated on the opposing ends. A first piece of evidence for this approach might be found in the fundamental difference in distribution between the subscales. In our study, the IC subscale skews towards high values and the PM subscale towards low values. But for CMS, the majority of parents indicated middle values. This suggests that there might be a fundamental difference in how the scaling of the three subscales works. As such, a more appropriate way for future research to analyze CMS might be an unfolding model.

Conclusion

We propose a 3-factor, 16-item model for the German adaptation of the PRFQ. We managed to show good to excellent fit, concurrent validity, and good reliability for the CMS subscale, while the reliability of the IC and PM subscale needs further assessment and improvement. The 16-item German PRFQ has been proven a valid and promising self-report measure for future studies into attachment and psychopathology. Given the complex nature of mentalizing and PRF, we encourage further validation studies of our German version of the PRFQ by other independent research groups.

Acknowledgements

We would like to thank all volunteers who participated in the online survey and all colleagues who contributed to this study and article.

Conflict of interest

The authors declare that there were no conflicts of interest with respect to the authorship or the publication of this article.

Publication Ethics

The study had been approved by the independent ethics committee of the medical faculty, Ruprecht-Karls-Universität, Heidelberg, in agreement with the ethics committee of the Ludwig-Maximilians-Universität, Munich (vote: S-446/2017) and the declaration of Helsinki 2013, seventh revision.

Authorship

Andreas S. Wildner, conceptualization, methodology, analysis, investigation, data curation, original draft and visualization; Su Mevsim Küçükakyüz, methodology, analysis, investigation, data curation, original draft, visualization; Anton K. G. Marx, methodology, investigation, data curation, review & editing; Tobias Nolte, conceptualization, review & editing; Corinna Reck, conceptualization, review & editing; Peter Fonagy, conceptualization, review & editing; Patrick Luyten, conceptualization, review & editing; Alexandra von Tettenborn, investigation, review & editing; Mitho Müller, methodology, review & editing; Anna-Lena

Zietlow, conceptualization, review & editing; Christian F. J. Woll, conceptualization, methodology, formal analysis, investigation, original draft, visualization, supervision

Open Data

Supplementary data to this article can be found online at [insert link of journal here]. The hypotheses, analysis plan, and cut-off criteria used in this study have been preregistered and are available along with the figures, analysis script, and all raw data necessary to reproduce the reported findings, under https://osf.io/j69wx/.

Funding

This research did not receive any specific grant from funding agencies in the public, commercial, or not-for-profit sectors.

Literature Cited

1. Luyten P, Nijssens L, Fonagy P, Mayes LC. Parental Reflective Functioning: Theory, Research, and Clinical Applications. The Psychoanalytical Study of the Child 2017; 70(a):174–99.

2. Fonagy P, Gergely G, Jurist E, Target M. Affect regulation, mentalization, and the development of the self. New York: Other Press; 2002.

3. Luyten P, Mayes LC, Nijssens L, Fonagy P. The parental reflective functioning questionnaire: Development and preliminary validation. PLoS ONE 2017; 12(5).

4. Rutherford HJ, Booth CR, Luyten P, Bridgett DJ, Mayes LC. Investigating the association between parental reflective functioning and distress tolerance in motherhood. Infant Behavior and Development 2015; 40:54–63.

5. Rutherford HJ, Goldberg B, Luyten P, Bridgett DJ, Mayes L.C. Parental reflective functioning is associated with tolerance of infant distress but not general distress: evidence for a specific relationship using a simulated baby paradigm. Infant Behavior and Development 2013; 36(4):635–41.

6. Burkhart ML, Borelli JL, Rasmussen HF, Brody R, Sbarra DA. Parental mentalizing as an indirect link between attachment anxiety and parenting satisfaction. Journal of Family Psychology 2017; 31(2):203–13.

7. Nijssens L, Luyten P, Malcorps S, Vliegen N, Mayes L. Parental reflective functioning and theory of mind acquisition: A developmental perspective. British Journal of Developmental Psychology 2021; 39(2):584–602.

8. Fonagy P, Luyten P, Allison E. Epistemic petrification and the restoration of epistemic trust: a new conceptualization of borderline personality disorder and its psychosocial treatment. Journal of Personality Disorders 2015; 29(5):575–609.

9. Fonagy P, Allison E. The role of mentalizing and epistemic trust in the therapeutic relationship. Psychotherapy 2014; 51(3):372–80.

10. Meins E, Fernyhough C, Fradley E, Tuckey M. Rethinking maternal sensitivity: Mothers' comments on infants' mental processes predict security of attachment at 12 months. The Journal of Child Psychology and Psychiatry and Allied Disciplines 2001; 42(5):637–48.

11. Slade A, Grienenberger J, Bernbach E, Levy D, Locker A. Maternal reflective functioning, attachment, and the transmission gap: A preliminary study. Attachment & Human Development 2005; 7(3):283–98.

12. Malcorps S, Vliegen N, Fonagy P, Luyten P. A four-year multi-wave prospective study on the role of parental reflective functioning and parenting stress in the development of socio-emotional problems in internationally adopted children. Development and Psychopathology 2024; 36(1):266–79.

13. Kristen K, Arietta S, Grienenberger JF. Maternal reflective functioning, mother–infant affective communication, and infant attachment: Exploring the link between mental states and observed caregiving behavior in the intergenerational transmission of attachment. Attachment & Human Development 2005; 7(3):299–311.

14. Salo SJ, Pajulo M, Vinzce L, Raittila S, Sourander J, Kalland M. Parent Relationship Satisfaction and Reflective Functioning as Predictors of Emotional Availability and Infant Behavior. Journal of Child and Family Studies 2021; 30:1214–28.

15. Sharp C, Fonagy P. The parent's capacity to treat the child as a psychological agent: constructs, measures and implications for developmental psychopathology. Social Development 2008; 17(3):737–54.

16. Duschinsky R, Foster S. Mentalizing and epistemic trust: The work of Peter Fonagy and colleagues at the Anna Freud Centre. New York: Oxford University Press; 2021.

17. Krink S, Muehlhan C, Luyten P, Romer G, Ramsauer B. Parental reflective functioning affects sensitivity to distress in mothers with postpartum depression. Journal of Child and Family Studies 2018; 27(5):1671–81.

18. Ainsworth MDS. Maternal sensitivity scales. Power 1969; 6:1379–88.

19. Fonagy P, Target M, Steele H, Steele M. Reflective-functioning manual version 5.0 for application to adult attachment interviews. London: University College London; 1998.

20. Roo M de, Wong G, Rempel GR, Fraser SN. Advancing Optimal Development in Children: Examining the Construct Validity of a Parent Reflective Functioning Questionnaire. JMIR Pediatrics and Parenting 2019; 2(1).

21. Lee Y, Meins E, Larkin F. Translation and preliminary validation of a Korean version of the parental reflective functioning questionnaire. Infant mental health journal 2021; 42:47–59.

22. Moreira H, Fonseca A. Measuring Parental Reflective Functioning: Further Validation of the Parental Reflective Functioning Questionnaire in Portuguese Mothers of Infants and Young Children. Child Psychiatry & Human Development 2022:1–13.

23. Pajulo M, Tolvanen M, Pyykkönen N, Karlsson L, Mayes L, Karlsson H. Exploring parental mentalization in postnatal phase with a self-report questionnaire (PRFQ): Factor structure, gender differences and association with sociodemographic factors. The Finn Brain Birth Cohort Study. Psychiatry Research 2018; 262:431–9.

24. Pazzagli C, Delvecchio E, Raspa V, Mazzeschi C, Luyten P. The Parental Reflective Functioning Questionnaire in Mothers and Fathers of School-Aged Children. Journal of Child and Family Studies 2017; 27:80–90.

25. Szabó B, Miklósi M, Boda M, Futó J. The adaptation of The parental reflective functioning questionnaire adolescent version to the Hungarian language and presentation of its psychometric characteristics. European Psychiatry 2022; 65(S1):207–8.

26. Ye P, Ju J, Zheng K, Dang J, Bian Y. Psychometric Evaluation of the Parental Reflective Functioning Questionnaire in Chinese Parents. Frontiers in Psychology 2022; 13.

27. Szabó B, Futó J, Luyten P, Boda M, Miklósi M. The psychometric properties of the Hungarian Parental Reflective Functioning Questionnaire. European journal of developmental psychology 2024; 21(1):106–23.

28. Bahramkhani M, Mohammad Hoseini S, Sarichloo ME. Factor Structure of Parental Reflective Functioning Questionnaire for Mothers Working at Qazvin University of Medical Sciences. Iran J Psychiatry Behav Sci 2024; 18(1).

29. Georg AK, Schröder-Pfeifer P, Cierpka M, Taubner S. Maternal parenting stress in the face of early regulatory disorders in infancy: a machine learning approach to identify what matters most. Frontiers in psychiatry 2019; 12.

30. Mattheß J, Eckert M, Richter K, Koch G, Reinhold T, Vienhues P et al. Efficacy of Parent-Infant-Psychotherapy with mothers with postpartum mental disorder: study protocol of the randomized controlled trial as part of the SKKIPPI project. Trials 2020; 21(1):1–12.

31. Griffee DT. Questionnaire Translation and Questionnaire Validation: Are They the Same? In: St. Louis, MO: Annual Meeting of the American Association of Applied Linguistics; 2001.

32. Campbell C, Tanzer M, Saunders R, Booker T, Allison E, Li E et al. Development and validation of a self-report measure of epistemic trust. PLoS ONE 2021; 16(4).

33. Zietlow A-L, Woll CFJ, Nonnenmacher N, Müller M, Labonte V, Ditzen B et al. Study protocol of the COMPARE-Interaction study: the impact of maternal comorbid depression and anxiety disorders in the peripartum period on child development. BMJ Open 2022; 12(1):e050437.

34. Wildner AS, Küçükakyüz SM, Woll CFJ. PRFQ (GER) Validation; 2023. Available from: URL: https://osf.io/j69wx/.

35. Reis D, Lehr D, Heber E, Ebert DD. The German Version of the Perceived Stress Scale (PSS-10): Evaluation of Dimensionality, Validity, and Measurement Invariance With Exploratory and Confirmatory Bifactor Modeling. Assessment 2019; 26(7):1246–59.

36. Bergant A, Nguyen T, Heim K, Ulmer H, Dapunt O. Deutschsprachige Fassung und Validierung der »Edinburgh postnatal depression scale«. DMW-Deutsche Medizinische Wochenschrift 1998; 123(3):35–40.

37. Cox JL, Holden JM, Sagovsky R. Detection of postnatal depression. Development of the 10-item Edinburgh Postnatal Depression Scale. The British Journal of Psychiatry: The Journal of Mental Science 1987; 150:782–6.

38. Haldweg K. Fragebogen zur Partnerschaftsdiagnostik (FPD). Handanweisung. Hogrefe Verlag für Psychologie; 1996.

39. R Core Team. R: A Language and Environment for Statistical Computing. Vienna, Austria; 2021. Available from: URL: http://www.R-project.org/.

40. Rosseel Y. lavaan: An R Package for Structural Equation Modeling. Journal of Statistical Software 2012; 48(2):1–36.

41. Wickham H, Averick M, Bryan J, Chang W, McGowan LD, François R et al. Welcome to the tidyverse. Journal of Open Source Software 2019; 4(43):1686.

42. Becker JP, Knowles JE. Convenience Functions for Education Data; 2020. Available from: URL: https://cran.r-project.org/web/packages/eeptools/eeptools.pdf.

43. Grolemund G, Wickham H. Dates and Times Made Easy with lubridate. Journal of Statistical Software 2011; 40(3):1–25. Available from: URL: https://www.jstatsoft.org/v40/i03/.

44. Revelle R. psych: Procedures for Psychological, Psychometric, and Personality Research. Evanston, Illinois; 2022. Available from: URL: https://CRAN.R-project.org/package=psych.

45. Tierney N, Cook D, McBain M, Fay C, O´Hara-Wild M, Hester J et al. Data Structures, Summaries, and Visualisations for Missing Data; 2021. Available from: URL: https://cran.r-project.org/web/packages/naniar/naniar.pdf.

46. Epskamp S, Stuber S, Nak J, Veenman M, Jorgensen TD. Path Diagrams and Visual Analysis of Various SEM Packages' Output; 2022. Available from: URL: https://cran.r-project.org/web/packages/semPlot/semPlot.pdf.

47. Zhang Z, Yuan KH. Robust Coefficient Alpha and Omega with Missing and Non-Normal Data; 2020. Available from: URL: https://cran.r-project.org/web/packages/coefficientalpha/coefficientalpha.pdf.

48. Harell FE. Harrell Miscellaneous; 2022. Available from: URL: https://cran.r-project.org/web/packages/Hmisc/Hmisc.pdf.

49. Komsta L. Moments, Cumulants, Skewness, Kurtosis and Related Tests; 2022. Available from: URL: https://cran.r-project.org/web/packages/moments/moments.pdf.

50. Meyer D, Dimitriadou E, Hornik K, Weingessel A, Leisch F, Chang CC et al. Misc Functions of the Department of Statistics, Probability Theory Group (Formerly: E1071), TU Wien; 2022. Available from: URL: https://cran.r-project.org/web/packages/e1071/e1071.pdf.

51. Jackson DL, Gillaspy JA, Purc-Stephenson R. Reporting practices in confirmatory factor analysis: An overview and some recommendations. Psychological Methods 2009; 14(1):6–23.

52. McDonald RP. Test Theory: A Unified Treatment. Mahwah, NJ: Erlbaum; 1999.

53. Cronbach LJ. Coefficient alpha and the internal structure of tests. Psychometrika 1951; 16(3):297–334.

54. Bland JM, Altman DG. Statistics notes: Cronbach's alpha. BMJ (Clinical Research Ed.) 1997; 314(7080):572.

55. Hu L, Bentler PM. Cutoff criteria for fit indexes in covariance structure analysis: Conventional criteria versus new alternatives. Structural Equation Modeling: A Multidisciplinary Journal 1999; 6(1):1–55.

56. Kyriazos TA. Applied Psychometrics: Sample Size and Sample Power Considerations in Factor Analysis (EFA, CFA) and SEM in General. Psychology 2018; 9:2207–30.

57. Hoyle RH, editor. Handbook of Structural Equation Modeling. New York: Guilford Press; 2014.

58. Zwick WR, Velcer WF. Factors influencing five rules for determining the number of components to retain. Psychological Bulletin 1986; 99:432–42.

59. Cohen J. A power primer. Psychological Bulletin 1992; 112(1):155–9.

60. Waller NG. Direct Schmid-Leiman Transformations and Rank-Deficient Loadings Matrices. Psychometrika 2018; 83(4):858–70.

61. Hair JF, Tatham RL, Anderson RE, Black W. Multivariate data analysis. 5th ed. London: Pentice-Hall; 1998.

62. Müller S, Wendt LP, Zimmermann J. Development and Validation of the Certainty About Mental States Questionnaire (CAMSQ): A Self-Report Measure of Mentalizing Oneself and Others. Assessment 2023; 30(3):651–74.

63. Carlone C, Milan S, Decoste C, Borelli JL, McMahon TJ, Suchman NE. Self-reportmeasure of parental reflective functioning: A study of reliability and validity across three samples of varying clinical risk. Infant mental health journal 2023; 00:1–15.

64. Slade A. Parental reflective functioning: An introduction. Attachment & Human Development 2005; 7(3):269–81.

65. Bo S, Sharp C, Fonagy P, Kongerslev M. Hypermentalizing, attachment, and epistemic trust in adolescent BPD: Clinical illustrations. Personality Disorders: Theory, Research, and Treatment 2017; 8(2):172.

66. Schulreich S, Tusche A, Kanske P, Schwabe L. Altruism under Stress: Cortisol Negatively Predicts Charitable Giving and Neural Value Representations Depending on Mentalizing Capacity. Journal of Neuroscience 2022; 42(16):3445–60. Available from: URL: https://www.jneurosci.org/content/42/16/3445.

67. Woll CFJ. Mother-infant relationship, dyadic interaction, and child development: the role of maternal mental health. Dissertation, LMU München: Fakultät für Psychologie und Pädagogik; 2023.

68. Klein EM, Brähler E, Dreier M, Reinecke L, Müller KW, Schmutzer G et al. The German version of the Perceived Stress Scale – psychometric characteristics in a representative German community sample. BMC Psychiatry 2016; 16.

69. Maxwell SE, Michael YL, George SH. Is psychology suffering from a replication crisis? What does “failure to replicate” really mean? American Psychologist 2015; 70(6):487–98.

Click here to access/download
Supporting Information
PRFQ ESM.docx
